# COVID‐19 clinical outcomes and DMT of MS patients and population‐based controls

**DOI:** 10.1002/acn3.51646

**Published:** 2022-08-22

**Authors:** Elisa Longinetti, Hannah Bower, Kyla A McKay, Simon Englund, Joachim Burman, Katharina Fink, Anna Fogdell‐Hahn, Martin Gunnarsson, Jan Hillert, Annette Langer‐Gould, Jan Lycke, Petra Nilsson, Jonatan Salzer, Anders Svenningsson, Johan Mellergård, Tomas Olsson, Fredrik Piehl, Thomas Frisell

**Affiliations:** ^1^ Department of Clinical Neuroscience Karolinska Institutet Stockholm Sweden; ^2^ Clinical Epidemiology Division, Department of Medicine Solna Karolinska Institutet Stockholm Sweden; ^3^ Department of Neuroscience Uppsala University Uppsala Sweden; ^4^ Department of Neurology Örebro University Örebro Sweden; ^5^ Clinical and Translational Neuroscience Southern California Permanente Medical Group, Kaiser Permanente Los Angeles USA; ^6^ Department of Clinical Neuroscience University of Gothenburg Gothenburg Sweden; ^7^ Department of Clinical Sciences Division of Neurology, Lund University Lund Sweden; ^8^ Department of Clinical Sciences Neurosciences, Umeå University Umeå Sweden; ^9^ Department of Clinical Sciences Danderyd Hospital, Karolinska Institutet Stockholm Sweden; ^10^ Department of Biomedical and Clinical Sciences Linköping University Linköping Sweden

## Abstract

**Objective:**

To estimate risks for all‐cause mortality and for severe COVID‐19 in multiple sclerosis patients and across relapsing–remitting multiple sclerosis patients exposed to disease‐modifying therapies.

**Methods:**

We conducted a Swedish nationwide population‐based multi‐register linkage cohort study and followed all multiple sclerosis patients (*n* = 17,692 in March 2020), individually age‐, sex‐, and region‐matched to five population‐based controls (*n* = 86,176 in March 2020) during March 2020–June 2021. We compared annual all‐cause mortality within and across cohorts, and assessed incidence rates and relative risks for hospitalization, intensive care admission, and death due to COVID‐19 in relation to disease‐modifying therapy use, using Cox regression.

**Results:**

Absolute all‐cause mortality among multiple sclerosis patients was higher from March to December 2020 than in previous years, but relative risks versus the population‐based controls were similar to preceding years. Incidence rates of hospitalization, intensive care admission, and death due to COVID‐19 remained in line with those for all‐cause hospitalization, intensive care admission, and mortality. Among relapsing–remitting patients on rituximab, trends for differences in risk of hospitalization due to COVID‐19 remained in the demographics‐, socioeconomic status‐, comorbidity‐, and multiple sclerosis severity‐adjusted model.

**Interpretation:**

Risks of severe COVID‐19‐related outcomes were increased among multiple sclerosis patients as a whole compared to population controls, but risk increases were also seen for non‐COVID‐19 hospitalization, intensive care admission, and mortality, and did not significantly differ during the pandemic compared to pre‐pandemic years. The risk conveyed by disease‐modifying therapies was smaller than previously assumed, likely as a consequence of the possibility to better control for confounders.

## Introduction

The onset of the SARS‐CoV‐2 pandemic immediately raised concerns regarding its possible impact on individuals with multiple sclerosis (MS), who have higher morbidity and mortality than the general population.^1^ In addition, prior research had identified an increased risk for infection associated with certain MS disease‐modifying treatments (DMTs), likely attributable to their immunomodulatory effects.^2^, ^3^

For these reasons, major efforts were initiated in early 2020 to rapidly increase our understanding of how SARS‐CoV‐2 affects MS patients and to identify risk factors for adverse outcomes with COVID‐19. Initially, this was based on experiences from local cohorts and case series.^4^, ^5^, ^6^ Subsequently, several national and multinational collaborations were launched. Hence, a French MS cohort study, showed that age, disability level, and obesity were independent risk factors for severe COVID‐19.^7^ Similarly, a US cohort study, reported that ambulatory disability and older age were independent risk factors for worse COVID‐19, with additional risk factors comprising Black race, cardiovascular comorbidity, and recent treatment with corticosteroids.^4^ While the French study did not reveal any significant association between DMT exposure and COVID‐19 severity, such associations have been suggested in an Italian registry‐based study, where a composite measure of severe COVID‐19 (pneumonia, hospitalization, intensive care (ICU), and mortality) was increased two‐fold in MS patients on B cell‐depleting DMTs compared to other therapies.^8^ This was also corroborated in the US study, where exposure to rituximab or ocrelizumab increased the risk of hospitalization 4.5 and 1.63‐fold, respectively.^4^ Finally, more recent data from the international MS registry collaborative group, suggest that B cell‐depleting DMTs are associated with an approximately two‐fold increased risk of hospitalization and ICU admission compared to MS patients on other types of DMTs.^9^

While these efforts have provided valuable preliminary evidence, the validity, interpretation, and comparability of results might suffer from the selection of more severe cases and inadequate adjustment for confounding factors. In addition, to date, studies comparing COVID‐19 outcomes in relation to population‐based controls and to pre‐pandemic time periods are rare.

The objectives of this study were to: (1) Determine the mortality rate among patients with MS in relation to population‐based controls during the pandemic and pre‐pandemic period, (2) Assess incidence rates (IRs) and relative risks of COVID‐19‐related outcomes among patients with MS compared to the population‐based controls, and (3) Address the relation between use of rituximab compared to specific DMTs and COVID‐19 outcomes in unselected RRMS patients. To this end we linked data from the Swedish MS registry to a series of demographic and health care registries with virtually complete coverage, also allowing for matching to population‐based MS‐free individuals.

## Methods

### Setting

Communal spread of SARS‐CoV‐2 was declared by the Public Health Agency of Sweden in March 2020, however, unlike most other countries, no strict lockdown was instituted. By September 2020, the pandemic had resulted in 10,000 deaths^10^; one of the higher mortality rates at the time.^11^ During March 2020 to June 2021 three pandemic waves were identified in Sweden, the first from March 1, 2020, to September 30, 2020, and the second and third one lasting from October 1, 2020, to June 30, 2021.

Non‐legally binding general recommendations included working from home and social distancing but did not (until December 2020) include the use of face masks in crowded areas. In May 2020, the Public Health Agency of Sweden announced recommendations to exert special caution, including social distancing, for persons with neurological disabilities, however, no specific recommendations for persons with MS were issued. Due to emerging concerns about COVID‐19 risks associated with immunosuppressive therapies, treated individuals were recommended longer intervals between infusions, and were prioritized for SARS‐CoV‐2 vaccinations starting from April 2021, 1–2 months before the same age strata of the general population. As of June 30, 2021, 38% of the population aged 12 years and older was vaccinated with at least two doses, and 59% with one dose, using COVID‐19 vaccines Moderna, Novavax, Oxford/AstraZeneca, or Pfizer/BioNTechy.^12^, ^13^

### Study population

Through the unique personal identification numbers assigned to all Swedish residents, we cross‐linked the Swedish MS registry to the following demographic and health registries; Population,^14^ Migration,^14^ Cause of Death,^15^ Patient,^16^ Cancer,^17^ Prescribed Drug,^18^ Intensive Care,^19^ as well as the Longitudinal database for insurance and labor market studies.^20^ We used data from the Swedish MS registry^21^ to identify an open cohort of individuals, all prevalent MS patients from March 2015 to June 2021 (among these, *n* = 17,692 were alive and registered on March 1, 2020). Each individual was matched on year of birth, sex, and region of domicile, to five randomly selected population controls (*n* = 86,176 on March 1, 2020) from the Swedish Population Register, required to be alive and free from MS at the time their index individual was diagnosed with MS. A separate sub‐cohort of patients with relapsing–remitting MS (RRMS; *n* = 8,232) was created based on information on disease course and treatment status as of March 1, 2020, stratified into the following groups: dimethyl fumarate (*n* = 1,060), fingolimod (*n* = 657), injectables (*n* = 941, of which 69% interferon beta and 31% glatiramer acetate), natalizumab (*n* = 949), rituximab (*n* = 4,312), and teriflunomide treatment (*n* = 312). Consisting with national guidelines recommending treatment for MS patients with active disease,^22^ only about 3% of RRMS patients have no recorded DMT in the MS Registry (including patients with missing therapy data). Because 4% of RRMS patients changed DMT between March 2020 and June 2021, we recreated DMT cohorts defined by the treatment status on October 1, 2020, and identified 8,429 individuals on active treatment with dimethyl fumarate (*n* = 1073), fingolimod (*n* = 631), injectables (*n* = 923, of which 69% interferon beta and 31% glatiramer acetate), natalizumab (*n* = 1,036), rituximab (*n* = 4,447), or teriflunomide treatment (*n* = 319).

The study was approved by the Swedish Ethical Review Authority (2021–02384).

### Outcomes

We defined the following six outcomes; death from any cause (based on death certificates), death from COVID‐19 (based on main and contributory causes of death recorded on death certificates), hospitalization for any cause and due to COVID‐19 (primary and secondary International Classification of Disease codes in the Patient Register), and ICU admission for any cause and due to COVID‐19 (the Intensive Care Register).

### Covariates

The register linkage provided data on age, sex, region of domicile, and characteristics of the MS patients including MS severity scores (Expanded Disability Status Scale,^23^ EDSS, Symbol Digit Modalities Test,^24^ SDMT, the physical component of the MS Impact Scale,^25^ MSIS‐29) disease duration, DMT use, and the prevalence of specific comorbid conditions (including the history of hospitalizations), educational level, country of birth, and civil status at cohort entry (Table 1 and eTable 1 for definitions). All covariates were updated over time to reflect the status at the start of follow‐up, in each analysis. No imputation of missing data was performed.

**Table 1:** Characteristics of MS patients in Sweden and their age‐, sex‐, and region‐ matched population‐based controls as of March 1, 2015, March 1, 2019, and March 1, 2020.

	2015–2019*		2020	
	MS	Population‐based controls	MS	Population‐based controls
N	91,478	450,376	17,692	86,176
Demographics				
Age, median (IQR)	48 (37–60)	48 (37–59)	51 (40–62)	50 (39–61)
Female	70%	70%	70%	70%
MS duration years, median (IQR)	8.0 (2.0–16.0)	n/a	11.0 (5.0–19.0)	n/a
Comorbidities				
Cancer	2%	2%	3%	2%
Diabetes	5%	5%	6%	6%
Heart failure	1%	0%	1%	1%
Ischemic heart disease	1%	1%	2%	1%
Infection	5%	1%	5%	1%
Lung disease	4%	3%	4%	3%
Kidney failure	1%	1%	1%	1%
Stroke	2%	1%	2%	1%
Venous thromboembolism	0.5%	0.3%	0.6%	0.4%
Highest education level				
Elementary	3%	4%	3%	4%
High school	56%	54%	54%	53%
University	41%	42%	43%	44%
Civil status				
Married	45%	45%	46%	46%
Single	55%	55%	54%	54%
Country of birth				
Sweden	88%	83%	88%	83%
Europe	7%	8%	7%	8%
Outside Europe	5%	9%	5%	9%
Hospital days				
Previous year, median (IQR)	7 (3–17)	5 (2–13)	8 (3–14)	5 (2–13)
Previous 10 years, median (IQR)	8 (3–25)	4 (2–10)	8 (3–25)	4 (2–10)

IQR, interquartile range; MS, multiple sclerosis; n/a, not applicable.

^*^ Including all prevalent MS patients and population‐based controls for each year.

### Statistical analysis

To assess changes in absolute all‐cause mortality from March to December 2020 compared to previous years, we defined annual cohorts (2015 to 2019) of all prevalent individuals with MS, and of their matched population comparator subjects, who were followed from March 1 to December 31 each year, censored at emigration or death. For each cohort (MS and controls), we calculated monthly crude mortality rates and the corresponding averages for 2015 to 2019. We used Cox regression to estimate crude and adjusted relative risks (expressed as hazard ratios, HRs) comparing individuals with MS to the population‐based controls from March to December of each year 2015 through 2020 (Tables 2, 3 and eTable 1 for details). To test if the relative mortality rate was higher in 2020 than during 2015–2019 we applied an interaction term between indicator variables for year 2020, and for MS patients.

**Table 2:** All‐cause mortality March–December each year 2015 through 2020 among MS patients compared with their general population‐based controls through HRs from Cox regression.

			HR (95% CI)		
	Year	N of deaths in the MS cohort	Model 1	Model 2	P for interaction 2020 versus 2015–2019
MS	2015	120	2.30 (1.85, 2.86)	1.41 (1.13, 1.77)	
	2016	121	2.00 (1.62, 2.47)	1.20 (0.97, 1.50)	
	2017	143	2.53 (2.07, 3.10)	1.50 (1.21, 1.85)	
	2018	139	2.22 (1.82, 2.71)	1.31 (1.06, 1.62)	
	2019	156	2.46 (2.03, 2.98)	1.49 (1.22, 1.81)	
	2020	182	2.56 (2.13, 3.06)	1.52 (1.25, 1.84)	0.432
RRMS	2015	10	0.63 (0.33, 1.22)	0.54 (0.28, 1.04)	
	2016	17	1.01 (0.60, 1.70)	0.86 (0.51, 1.45)	
	2017	24	1.36 (0.86, 2.13)	1.16 (0.73, 1.85)	
	2018	24	1.31 (0.84, 2.05)	1.16 (0.73, 1.84)	
	2019	14	0.69 (0.39, 1.20)	0.59 (0.34, 1.04)	
	2020	30	1.30 (0.87, 1.95)	1.04 (0.69, 1.57)	0.409
PPMS	2015	27	2.76 (1.70, 4.46)	1.55 (0.94, 2.57)	
	2016	26	1.73 (1.09, 2.72)	0.98 (0.61, 1.56)	
	2017	38	3.75 (2.45, 5.72)	2.01 (1.31, 3.10)	
	2018	31	2.52 (1.63, 3.89)	1.30 (0.83, 2.04)	
	2019	33	2.70 (1.76, 4.15)	1.37 (0.87, 2.14)	
	2020	39	2.79 (1.88, 4.14)	1.47 (0.97, 2.24)	0.799
SPMS	2015	83	3.02 (2.29, 3.98)	1.58 (1.19, 2.09)	
	2016	78	2.60 (1.97, 3.43)	1.33 (0.99, 1.77)	
	2017	81	2.71 (2.06, 3.57)	1.38 (1.03, 1.84)	
	2018	84	2.51 (1.92, 3.27)	1.25 (0.95, 1.66)	
	2019	109	3.36 (2.63, 4.30)	1.73 (1.34, 2.24)	
	2020	113	3.16 (2.48, 4.02)	1.66 (1.29, 2.14)	0.430

**Model 1**: Adjusted for sex, age, region, and time from March 1 of each year.

**Model 2**: Further adjusted for country of birth, education, civil status, comorbidities, n of hospital days the previous year, n of hospital days previous 10 years.

CI, confidence interval; HR, hazard ratio; MS, multiple sclerosis; PPMS, primary progressive MS; RRMS, relapsing–remitting MS; SPMS, secondary progressive MS.

**Table 3:** Incidence rates and relative risks for COVID‐19‐related events and other outcomes in MS patients, compared with matched population‐based controls, March 1, 2020, through June 30, 2021.

Cohort	Outcome	Events, *n*	IR per 100,000 person‐years (95% CI)	HR (95% CI)	
				Model 1	Model 2
MS	Death, any	284	3.34 (2.97–3.75)	2.55 (2.21, 2.94)	1.53 (1.31, 1.79)
	Death, COVID‐19	32	0.38 (0.27–0.53)	2.56 (1.67, 3.92)	1.88 (1.18, 3.02)
	Hospitalization, any	3,720	46.3 (44.8–47.8)	1.95 (1.88, 2.02)	1.40 (1.34, 1.46)
	Hospitalization, COVID‐19	336	3.97 (3.57–4.42)	2.91 (2.54, 3.33)	2.36 (2.04, 2.72)
	ICU admission, any	179	2.11 (1.82–2.44)	2.26 (1.89, 2.70)	1.48 (1.22, 1.80)
	ICU admission, COVID‐19	40	0.47 (0.34–0.64)	3.00 (2.02, 4.45)	2.92 (1.94, 4.40)
Controls	Death, any	544	1.31 (1.20–1.43)	Ref.	Ref.
	Death, COVID‐19	61	0.15 (0.11–0.19)	Ref.	Ref.
	Hospitalization, any	9,560	23.7 (23.3–24.2)	Ref.	Ref.
	Hospitalization, COVID‐19	565	1.36 (1.26–1.48)	Ref.	Ref.
	ICU admission, any	387	0.93 (0.84–2.11)	Ref.	Ref.
	ICU admission, COVID‐19	65	0.16 (0.12–0.20)	Ref.	Ref.

**Model 1**: Adjusted for age, sex, region, and time from start of each pandemic wave.

**Model 2**: Further adjusted for country of birth, education, civil status, comorbidities, n of hospital days the previous year, n of hospital days previous 10 years.

CI, confidence interval; HR, hazard ratio; IR, incidence rate; MS, multiple sclerosis.

For each defined cohort, we next extended the follow‐up until June 30, 2021, and calculated crude IRs per 100,000 person‐years and relative risks for hospitalization, ICU admission, and death due to COVID‐19, during March 1, 2020, to June 30, 2021. We calculated relative risks via unadjusted and adjusted Cox models as described above. We also applied an additional model further adjusted for MS severity, disease duration, and number of previous DMTs. We used time since first pandemic wave (March 1, 2020) as the underlying time‐scale in all models and re‐started the time scale at beginning of the second pandemic wave (October 1, 2020) using a robust sandwich estimator to account for multiple data contribution from the study participants. To contextualize the COVID‐19‐related outcomes, we also assessed all‐cause hospitalization, ICU admission, and death.

To investigate the association between DMTs and each of the outcomes we compared patients exposed to rituximab to dimethyl fumarate, fingolimod, injectables, natalizumab, and teriflunomide separately, and fitted Cox regressions, progressively adjusted for age (modeled with third‐degree polynomial), sex, region of domicile, comorbidity, healthcare resource utilization, socioeconomic status, MS severity, disease duration, and number of previous DMTs.

To investigate the potential impact of cumulative rituximab doses, we stratified the rituximab cohort by number of rituximab doses received before March 1, 2020 (≤2 and ≥3). To investigate the potential impact of time since preceding dose of rituximab we stratified the rituximab cohort by time since preceding dose at the start of pandemic wave (<8 months and ≥8 months).

All analyses were made with Stata V.16.1 and SAS V.9.4.

## Results

### All‐cause mortality in MS patients and their matched population‐based controls compared to preceding years

**Figure 1:**
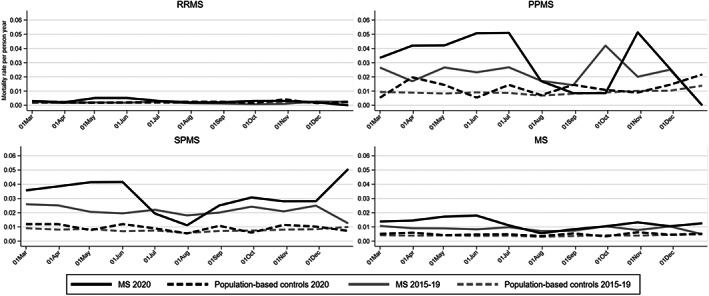
All‐cause mortality of MS patients in Sweden, and among individually matched population‐based controls, during March 1, 2020, until December 31, 2020, compared with the corresponding average mortality during the same seasons 2015 through 2019. MS, multiple sclerosis; PPMS, primary progressive MS; RRMS, relapsing–remitting MS; SPMS, secondary progressive MS.

We identified 17,692 MS patients alive and residing in Sweden on March 1, 2020 (Table 1, and by MS course in eTables 2–4), and by December 30, 2020 (14,686 person‐years), 180 (1.0%) of these patients had died. Figure 1 depicts the monthly mortality rate in the MS cohort, by clinical subtype, and in the population‐based controls during 2020 and the average mortality rate from 2015 to 2019. In all cohorts, the mortality during 2020 was higher than during previous years, except for primary progressive MS (PPMS) during August–October, and secondary progressive MS (SPMS) during July–August. There were also elevated unadjusted and adjusted annual HRs of all‐cause mortality in the MS cohort as a whole in each examined year (Table 2). Notably, however, an elevated risk was only evident in the progressive forms of MS, but not RRMS. In addition, the HR for 2020 did not deviate substantially from the corresponding HRs 2015 through 2019. Adjusting for comorbid conditions and socioeconomic status, reduced the HR in all categories, and PPMS no longer appeared to be associated with increased mortality relative to the population‐based controls.

### COVID‐19‐related and other hospitalization, ICU admission, and mortality among MS patients and population‐based controls during the pandemic waves

We followed the cohort of 17,692 MS patients from March 1, 2020, to June 30, 2021, for a total of 23,329 person‐years. Among all individuals with MS, IR of death per 100,000 person‐years from COVID‐19 was 0.38 (95% CIs 0.27–0.53) vs 0.15 (95% CIs 0.11–0.19) in controls, 3.97 (95% CIs 3.57–4.42) vs 1.36 (95% CIs 1.26–1.48) for hospitalization with COVID‐19, and 0.47 (0.34–0.64) vs 0.16 (95% CIs 0.12–0.20) for ICU admission due to COVID‐19 (Table 3). This could be contrasted to an IR of death from any cause of 3.34 (95% CIs 2.97–3.75) vs 1.31 (95% CIs 1.20–1.43) in controls, for hospitalization for any cause of 46.3 (95% CIs 44.8–47.8) vs 23.7 (95% CIs 23.3–24.2), for ICU admission for any cause of 2.11 (95% CIs 1.82–2.44) vs 0.93 (95% CIs 0.84–1.03; Table 3).

Relative risks for each of the studied outcomes were all elevated in the MS cohort with somewhat higher unadjusted HRs for the COVID‐19‐specific outcomes than those due to any cause (Table 3). However, adjustment for comorbidity and socioeconomic status lowered the associations between MS and all outcomes.

### COVID‐19‐related and other hospitalization, ICU admission, and mortality in relation to DMTs among RRMS patients

For this analysis, we followed RRMS patients exposed to dimethyl fumarate, fingolimod, injectables, natalizumab, rituximab, or teriflunomide from March 1, 2020, until September 30, 2020 (*n* = 8,232), and from October 1, 2020, until June 30, 2021 (*n* = 8,429) for a total of 17,206 person‐years (patient characteristics in eTables 5–6).

Using dimethyl fumarate as reference, the largest DMT group outside rituximab (see Table 4 for crude IRs and HRs), there was a higher risk of hospitalization (any cause and COVID‐19 specifically) associated with rituximab, but it decreased in magnitude and was not statistically significant after adjustment for comorbidity, socioeconomic status, and MS severity. Similarly, using fingolimod, natalizumab, or teriflunomide as reference, use of rituximab was associated with a higher risk of hospitalization (any cause and COVID‐19 specifically) which decreased in magnitude after adjustment for comorbidity, socioeconomic status, and MS severity. In contrast, when comparing rituximab use to injectables, the risk of hospitalization (any cause) increased after adjusting for MS severity. We observed two deaths due to COVID‐19, both among patients treated with rituximab, and 14 deaths due to any cause, among patients exposed to dimethyl fumarate (*n* = 2), fingolimod (*n* = 1), natalizumab (*n* = 3), and rituximab (*n* = 8; HRs not applicable due to low number of events).

**Table 4:** Occurrence, incidence rates, and relative risks of COVID‐19‐ and any cause‐related hospitalizations and ICU admission in RRMS patients, March 1, 2020, through June 30, 2021, according to DMT treatment status at the start of first (March 1, 2020, *n* = 8,232) and second pandemic wave (October 1, 2020, *n* = 8,429).

DMT	Outcome	Events, *n*	IR per 100,000 person‐years (95% CI)	Rituximab vs each DMT, HR (95% CI)		
				Model 1	Model 2	Model 3
Rituximab	Hospitalization, any	688	33.8 (31.3–36.4)			
	Hospitalization, COVID‐19	104	4.92 (4.06–5.97)			
	ICU admission, any	34	1.60 (1.14–2.24)			
	ICU admission, COVID‐19	15	0.71 (0.43–1.17)			
Dimethyl	Hospitalization, any	122	24.3 (20.4–29.0)	1.40 (1.14, 1.73)	1.23 (1.00, 1.51)	1.24 (0.99, 1.56)
fumarate	Hospitalization, COVID‐19	13	2.52 (1.46–4.34)	1.98 (1.10, 3.56)	1.79 (0.98, 3.25)	1.48 (0.80, 2.74)
	ICU admission, any	2	n/a	n/a	n/a	n/a
	ICU admission, COVID‐19	1	n/a	n/a	n/a	n/a
Fingolimod	Hospitalization, any	60	19.8 (15.4–25.5)	1.71 (1.30, 2.25)	1.47 (1.11, 1.93)	1.40 (1.05, 1.89)
	Hospitalization, COVID‐19	6	1.93 (0.89–4.30)	2.71 (1.20, 6.15)	2.41 (1.05, 5.54)	2.11 (0.92, 4.81)
	ICU admission, any	3	n/a	n/a	n/a	n/a
	ICU admission, COVID‐19	0	n/a	n/a	n/a	n/a
Injectables	Hospitalization, any	96	21.9 (17.9–26.7)	1.66 (1.31, 2.11)	1.51 (1.20, 1.91)	2.04 (1.52, 2.75)
	Hospitalization, COVID‐19	3	n/a	n/a	n/a	n/a
	ICU admission, any	3	n/a	n/a	n/a	n/a
	ICU admission, COVID‐19	0	n/a	n/a	n/a	n/a
Natalizumab	Hospitalization, any	95	20.1 (16.4–24.6)	1.65 (1.30, 2.09)	1.59 (1.26, 2.01)	1.49 (1.17, 1.90)
	Hospitalization, COVID‐19	7	1.45 (0.69–3.05)	3.16 (1.46, 6.83)	2.95 (1.37, 6.36)	2.73 (1.23, 6.05)
	ICU admission, any	6	1.24 (0.56–2.77)	1.08 (0.44, 2.69)	1.17 (0.47, 2.88)	1.26 (0.47, 3.42)
	ICU admission, COVID‐19	3	n/a	n/a	n/a	n/a
Teriflunomide	Hospitalization, any	25	16.6 (11.2–24.6)	2.14 (1.42, 3.23)	1.86 (1.25, 2.78)	1.70 (1.13, 2.57)
	Hospitalization, COVID‐19	4	n/a	n/a	n/a	n/a
	ICU admission, any	2	n/a	n/a	n/a	n/a
	ICU admission, COVID‐19	1	n/a	n/a	n/a	n/a

**Model 1**: Adjusted for age, sex, region, and time from the start of each pandemic wave.

**Model 2**: Further adjusted for country of birth, education, civil status, comorbidities, n of hospital days the previous year, n of hospital days previous 10 years.

**Model 3**: Further adjusted for EDSS, SDMT, physical component of MSIS‐29, MS duration, n previous DMTs.

CI, confidence interval; DMT, disease‐modifying therapy; EDSS, Expanded Disability Status Scale; ICD, International Classification of Disease; IR, incidence rate; HR, hazard ratio; MSIS, Multiple Sclerosis Impact Scale; MS, Multiple Sclerosis; n/a, not applicable due to low number of events (<5); RRMS, relapsing–remitting MS; SDMT, Symbol Digit Modalities Test.

Exposure to a higher number of prior rituximab doses (≤2 vs ≥3) was not associated with higher risks (eTables 7–8), nor was exposure to a shorter time (<8 months vs ≥8 months) between preceding rituximab infusion at pandemic wave start (eTables 9–10).

## Discussion

We report hospitalization, ICU admission, and mortality rates among MS patients compared with matched controls in a nationwide study set during the COVID‐19 pandemic. Importantly, we also had the opportunity to compare the rates recorded during the pandemic with those of the preceding years. From March–December 2020, MS patients experienced approximately 50% higher mortality from any cause compared to the population‐based controls. However, this increase was of a similar magnitude in the years preceding the pandemic, and could largely be explained by comorbidity and socioeconomic factors. In absolute terms, the risks for patients with MS compared to MS‐free controls regarding adverse COVID‐19 outcomes translated into an additional 2.61 cases of hospitalization per 100,000 person‐years, an additional 0.31 cases of ICU admission per 100,000 person‐years, and an additional 0.23 cases of death per 100,000 person‐years. It is important to contextualize this in terms of the generally increased risk for all‐cause hospitalization, ICU admission, and death evident through all studied years.

Our observation that excess mortality during the pandemic was evident among patients with progressive disease alone, is in agreement with previous studies, with an emerging picture that among MS patients, much of the risk can be explained by age, disability status, comorbidity, and socioeconomic status. These observations are relevant for patient counseling because they suggest that for a person with MS, general health status seemed more important for COVID‐19 risk than MS itself. This underscores the importance of optimizing the treatment of comorbidity and promoting a healthy lifestyle among MS patients.

Our findings regarding the impact of MS DMTs are in line with previous reports suggesting a risk increase of rituximab use for COVID‐19 hospitalization in relation to other MS DMTs.^9^ In the present analysis, the risk increased of hospitalization for any cause associated with rituximab use were higher when using injectables as a reference, which would fit the perception that these DMTs are a generally safer option. In contrast to existing data,^9^ however, the risk increase for adverse COVID‐19 outcomes among individuals on rituximab was modest. In addition to lack of power due to low number of events, the fact that numerical risk estimates were lowered in the adjusted models suggest that incomplete adjustment for confounders may explain part of the difference between our results and the previous literature. Data relying on spontaneous reporting is also prone to surveillance bias, where individuals on high efficacy therapies requiring regular infusions may be followed more carefully than those on oral or injectable DMTs. However, individuals treated with rituximab displayed higher point estimates for the risk of hospitalization (although not statistically significant in the fully adjusted model using dimethyl fumarate or fingolimod as reference), suggesting that B cell‐depletion is associated with some degree of risk increase. It should be noted that the rituximab treatment protocol for MS in Sweden^26^ involves lower doses than most other published protocols and that the suggestion of extending the interval between rituximab infusions during the pandemic might have further contributed to reducing possible risks associated with rituximab treatment. Further studies will be needed to address a possible relation between time since infusion of B cell‐depleting therapies and COVID‐19 outcomes, as well as impact on vaccination responses.

This study has certain limitations. We assessed risks for outcomes of known COVID‐19 cases, but we could not study risks for acquiring SARS‐CoV‐2 infection, nor a potentially negative interaction between SARS‐CoV‐2 infection and recent steroid treatment. We were also unable to test for a potentially negative interaction between SARS‐CoV‐2 infection and degree of B‐cell depletion and lymphopenia among patients treated with rituximab and fingolimod. Further, we adjusted for a number of possible confounders but lacked information on certain known COVID‐19 risk factors such as body mass index, hypertension, smoking, COVID‐19 vaccination status, and immune response, and therefore cannot rule out residual confounding. However, the vaccination rate was low during the study period^13^ (which was dominated by 2020, with no vaccinations), and will not have had a major impact on the incidence rate of infection or contrast across DMTs. Treatment status was as defined in the Swedish MS registry at the beginning of each pandemic wave, and thus subject to misclassification by patient‐ or physician‐initiated changes in response to the pandemic, though we note that there were few recorded treatment switches during follow‐up and that the expected duration of treatment effects should cover most of this time. Finally, due to the varying COVID‐19 incidence during the study period, some of the risk estimates might have been diluted.

Our study also has strengths, including that it involved virtually all MS patients and all DMT‐treated patients with RRMS nationwide, which could be observed years prior to the start of the first to the end of the third wave. Outcomes were determined independently of MS status, allowing for estimation of both absolute and relative risks compared to controls from the general population.

## Conclusions

We found that the risk magnitude for adverse COVID‐19 outcomes among MS patients in relation to MS‐free controls largely mirror those seen for all‐cause hospitalization, ICU admission, and mortality, with risk largely being explained by confounders rather than the diagnosis of MS per se. Differences in risk associated with the use of rituximab compared to specific DMTs were smaller than in previous studies, again likely explained by better control of confounding factors. Taken together this information is of value for conducting more precise benefit–risk evaluations with different interventions in the context of the COVID‐19 pandemic. Follow‐up studies are needed to address the effect of Sars‐CoV‐2 vaccinations among MS patients and in relation to different MS DMTs.

## Author Contributions

Conception and design of the study: EL, HB, FP, TF; acquisition of data: SE, JB, KF, AFH, MG, JH, ALG, JL, PN, JS, AS, JM, TO, FP, TF; analysis of data: EL; drafting a significant portion of the manuscript or figures: EL, HB, KAM, SE, JB, KF, AFH, JL, PN, JS, AS, JM, TO, FP, TF.

## Conflicts of Interest

EL, HB, KAM, SE, JB, MG, AS, TF reports no disclosure. KF has received lecture honoraria from Biogen, Novartis, Merck, and Roche. AFH has received unrestricted funding from Biogen Idec, Pfizer, Orion Pharma, and Celltrion, speaking honoraria from Merck, and consulting fee from Roche. JH has received travel support and/or lecture honoraria from Biogen, Novartis, Merck, Roche, Axelion, Sanofi, and BMS; has served on scientific advisory boards for Almirall, Biogen, Novartis, Merck, Roche, BMS, and Sanofi; serves on the editorial board of the Acta Neurologica Scandinavica; has received unconditional research grants from Biogen and Novartis. ALG receives grant support and awards from the Patient‐Centered Outcomes Research Institute and the National MS Society; she currently serves as a voting member on the California Technology Assessment Forum, a core program of the Institute for Clinical and Economic Review (ICER); she has received sponsored and reimbursed travel from ICER and the National Institutes of Health. JL has received travel support and/or lecture honoraria from Biogen, Novartis, Merck, Alexion, and Sanofi Genzyme; has served on scientific advisory boards for Almirall, Teva, Biogen, Novartis, Merck, Roche, Sanofi Genzyme, and BMS; serves on the editorial board of the Acta Neurologica Scandinavica; and has received unconditional research grants from Biogen, and Novartis. PN has received travel support from Bayer Schering Pharma, Merck Serono, Biogen, and Genzyme a Sanofi Company, honoraria for lectures and advisory boards from Merck Serono and Genzyme a Sanofi Company, advisory boards for Novartis and Roche, lectures for Biogen and has received unrestricted grants from Biogen. JS has received institutional consultancy fee from Mabion S.A. JM has received honoraria for Advisory boards for Sanofi Genzyme and Merck and lecture honorarium from Merck. TO has received advisory board/lecture compensations as well as unrestricted MS research grants from Biogen, Novartis, Merck, and Sanofi, and MS research grants from the Swedish Research Council and the Knut and Alice Wallenbeg Foundation. FP has received research grants from Merck KGaA and UCB, fees for serving on DMC in clinical trials with Chugai, Lundbeck, and Roche.
